# Functional Analysis of the *NinaB-like* Gene in Body Color Regulation of *Neocaridina denticulata sinensis*

**DOI:** 10.3390/biotech15010015

**Published:** 2026-02-05

**Authors:** Haifan Li, Lili Zhang, Guodong Wang, Tanjun Zhao

**Affiliations:** 1 Key Laboratory of Healthy Mariculture for the East China Sea, Ministry of Agriculture and Rural Affairs, Fisheries College, Jimei University, 43 Yindou Road, Jimei, Xiamen 361021, China; 19396365986@163.com (H.L.); llzhang@jmu.edu.cn (L.Z.); zhaotanjun233@163.com (T.Z.); 2 State Key Laboratory of Mariculture Breeding, Fisheries College, Jimei University, Xiamen 361021, China

**Keywords:** carotenoid accumulation, RNAi, SNP genotyping, chromatophore

## Abstract

Carotenoid-based pigmentation is crucial for the ornamental and commercial value of the cherry shrimp (*Neocaridina denticulata sinensis*). While several genes are known to influence carotenoid metabolism, the genetic basis for specific color strains remains largely unexplored. Here, we functionally characterized *NinaB-like*, a homolog of a carotenoid oxygenase, in cherry shrimp pigmentation. We employed qPCR to gain gene expression profiles, utilized RNAi technology to analysize the relation between its expression level and carotenoid accumulation, and performed GT-seq to identify genotypes of different color strains. Significant differential expression of *NinaB-like* was observed not only across distinct color strains but also during embryonic development of cherry shrimp (*p* < 0.05), peaking at the red strain and post-larval stage of cherry shrimp. RNA interference-mediated knockdown of *NinaB-like* resulted in a marked increase in red pigment deposition at the metanauplius and pre-zoea stages, confirming its role as a negative regulator of carotenoid accumulation. Importantly, we identified two tightly linked, non-synonymous SNPs (927C > A and 935A > C) within the *NinaB-like* coding region that exhibited a strong association with body color. Our study provides the first functional evidence that *NinaB-like* is a negative regulator of carotenoid degradation and a major genetic determinant for body color in cherry shrimp, providing new insights for genetic breeding and biological research.

## Simple Summary

NinaB-like, a homolog of a carotenoid oxygenase, is a negative regulator of carotenoid accumulation and a potential genetic determinant of body color in cherry shrimp (*Neocaridina denticulata sinensis*). Two non-synonymous SNPs in *NinaB-like* were associated with body color. This study provides a potential genetic manipulation site for cherry shrimp breeding.

## 1. Introduction

Coloration is a fascinating phenotypic trait in aquatic animals, and it participates in diverse ecological and physiological processes such as predator avoidance, mate selection, inter-specific communication, and environmental adaptation [1]. For crustaceans, one of the most diverse groups of aquatic invertebrates, their body color is primarily derived from carotenoids [2]. Carotenoid cleavage dioxygenases (CCDs) are key enzymes in carotenoid metabolic pathway, in which beta-carotene 15,15′-monooxygenase (BCO1) catalyzes carotenoids to produce retinal (vitamin A aldehyde) [3]. Although the functions of BCO1 have been extensively studied in mammals [4], its roles in regulating carotenoid metabolism and coloration in crustaceans remain insufficiently explored.

Cherry shrimp (*Neocaridina denticulata sinensis*) are a small freshwater ornamental shrimp that has gained widespread popularity among aquarium enthusiasts due to its vibrant coloration, short reproductive cycle, and high offspring survival rate [5]. The wild-type cherry shrimp typically has a transparent body with scattered black spots, and natural mutations have given rise to multiple stable color morphs—among which the red, yellow, and blue variants are the most economically important and widely cultivated in the ornamental shrimp industry [6]. Since the 1990s, artificial breeding of red mutant cherry shrimp has been carried out in Taiwan, China successfully [6].

Our research group previously conducted targeted metabolite analysis of carotenoids in cherry shrimp, revealing a significant correlation between body color phenotypes and carotenoid content [7]. However, the genetic basis underlying the formation of different color strains, especially the role of carotenoid metabolic enzymes such as *BCO1* homologs in regulating body color, remains largely unclear. Therefore, exploring the functional genes involved in carotenoid metabolism and color regulation in cherry shrimp is of great significance for elucidating the molecular mechanisms of crustacean coloration and promoting the genetic breeding of ornamental shrimp.

We identified a *BCO1* homolog in cherry shrimp via transcriptome analysis, which showed high similarity to *Drosophila NinaB* [8], hence named *NinaB-like* (GenBank Accession Number: PRJNA1209659). Given BCO1′s critical role in carotenoid cleavage and the close correlation between carotenoid content and body color in cherry shrimp, exploring *NinaB-like* function will fill the knowledge gap in crustaceans.

In this paper, we obtained the expression profile of *NinaB-like* in different color strains and development stages using qPCR. Cherry shrimp embryos were treated with *NinaB-like* RNAi. Two SNPs of *NinaB-like* were identified, and their correlation with body color was analyzed. The purpose of this study is to understand the role of *NinaB-like* in the pigmentation of cherry shrimp. This study provides functional evidence that *NinaB-like* is a key negative regulator of carotenoid degradation and a major genetic determinant of body color in cherry shrimp.

## 2. Materials and Methods

### 2.1. Shrimp Cultivation

Laboratory-maintained red, blue, yellow, and wild shrimp strains were used in this study [9]. These strains, bred for over three years, display genetically stable and strain-specific body coloration. The experimental animals were raised in glass tanks (40 cm × 30 cm × 25 cm). Aqua Design Amano (ADA) aquarium soil and *Elodea nuttallii* were added to the glass tank to build a micro-ecosystem. Water quality was maintained by the addition of an appropriate number of nitrifying bacteria (*Nitrosomonas* + *Nitrobacter*). Using aerated tap water for 24 h, a 50% water change was performed every three days, and the tank was emptied once a month. During the cultivation period, water temperature was controlled at 20–26 °C, pH was maintained at approximately 7.0–8.4, and dissolved oxygen was maintained at 7.7 mg/L. To maintain genetic isolation between strains, each strain was cultured in a separate aquarium. A total of 30 sexually mature females and 10 males were introduced into each tank. Following three months of culture, approximately 300 offspring were produced. The offspring were then evenly divided into three groups and transferred to separate tanks for continued cultivation. All experimental shrimps in subsequent experiments were sexually mature adults (3 months old after hatching) with consistent morphometric traits: body length 1.0–1.5 cm, body weight 0.10–0.15 g, and stable color phenotypes.

### 2.2. Tissues and Different Developmental Embryos Stages Collection

The adult females were immobilized by immersion in sterile water pre-cooled to 4 °C for 10 min until movement ceased. Tissue samples were obtained from seven individual shrimps, with each individual regarded as an independent biological replicate. Under aseptic conditions, dissections were performed in a sterile Petri dish using sterilized forceps to isolate distinct tissue samples. Micro-dissection was performed under a stereomicroscope (Motic China Group Co., Ltd., Hong Kong, China) using sterile micro-forceps and fine scissors. The following tissues were aseptically isolated in sequence from each individual: (1) compound eyes, excised at the optic stalk; (2) hepatopancreas, carefully separated from the midgut and surrounding connective tissue; (3) muscle tissue, collected from the abdominal segment; (4) exoskeletons, stripped from the dorsal cephalothorax region; (5) the entire digestive tract, dissected from the esophageal junction to the anus, with luminal contents gently flushed using chilled PBS (pH 7.4). All collected tissue samples were immediately flash-frozen in liquid nitrogen and stored at −80 °C before subsequent analysis.

To collect embryos, berried females (defined as individuals exhibiting externally attached eggs on their pleopods) were isolated and relocated to a separate tank. Each biological replicate consisted of all embryos originating from a same berried female (the number of embryos in each berried female is 30 to 50), and there were seven biological replicates for each developmental stage. The collect embryos were observed using a stereomicroscope to identify their developmental stages. Chromatophore initiates at the metanauplius stage and progresses through the pre-zoea and membrane-zoea stages. To investigate development profiles of target genes, five developmental stages were selected: pre-nauplius stage, metanauplius stage, pre-zoea stage, membrane-zoea stage, and post-larva stage. Embryonic staging followed the criteria outlined previously [10]. The storage method of the embryos was the same as the samples above.

### 2.3. Total RNA Extraction, cDNA Synthesis and qPCR Validation

Total RNA was extracted using RNA-solv reagent (Omega Bio-tek, Inc., Norcross, GA, USA) following the manufacturer’s instructions. The integrity and potential degradation of the extracted total RNA were assessed via 1% agarose gel electrophoresis. RNA samples were quantified and assessed for purity using a NanoBio100 micro ultraviolet spectrophotometer (OPTOSKY Co., Ltd., Beijing, China). Based on the resulting RNA concentrations, the appropriate template volume was calculated to ensure that 1 μg of total RNA was used for first-strand cDNA synthesis. Reverse transcription was performed using a cDNA synthesis kit (Tiangen Co., Ltd., Beijing, China). After synthesis, the cDNA products were diluted 10-fold and used as templates for subsequent qPCR analysis.

For the gene expression level of different color varieties, exoskeleton was selected as the research object tissue. Primers were designed using Primer3 software (version 3.0; https://sourceforge.net/projects/primer3/, accessed on 12 October 2023), and synthesized by TsingKe Biotechnology Co., Ltd. (Beijing, China). *Glyceraldehyde-3-phosphate dehydrogenase* (*GAPDH*; GenBank Accession Number: MZ734609) served as the reference gene for normalization [11]. Target gene expression was quantified using the 2^−ΔΔCt^ method [12]. Quantitative PCR (qPCR) reactions were performed following our previous report with modification in which annealing time was set for 30 s (at 60 °C) [13]. Differences between different groups were analyzed by one-way ANOVA followed by Tukey’s honestly significant difference (HSD) test for post hoc multiple comparisons [14].

### 2.4. Double-Stranded RNA (dsRNA) Generations

The dsRNA was accomplished according to the protocol described in our previous article [15]. A 200–500 bp region of ORF was selected as the template to transcript RNA for preparing dsRNA. The sequence of dsRNA template shows poor conservation, and this high sequence specificity minimized off-target effects. The template DNA fragments were amplified by PCR and purified using a PCR purification kit (Shanghai Generay Biotech Co., Ltd., Shanghai, China). The PCR product was identified using Sanger sequencing (Bo Rui Co., Ltd., Xiamen, China). The target DNA was subsequently ligated into the pGEM-T Easy Vector (Promega, San Luis Obispo, CA, USA). After ligation products transformed into DH5α competent cells, the insert orientation of positive colony was determined using PCR with a combination of sequence-specific primers (SP-F or SP-R) and a T7 promoter primer (T7-F). The two orientation colonies were selected for expansion, respectively. The plasmid DNA was extracted using a TIANGEN Plasmid Mini-Prep Kit (TIANGEN, Beijing, China). The sense line DNA template was generated via PCR amplification with a forward-oriented plasmid, T7 primer and SP-R primer, as well as an antisense line DNA template with a reverse-oriented plasmid, T7 primer and SP-F primer. In vitro transcription was performed using an in vitro transcription kit (Thermo Fisher Scientific, Waltham, MA, USA), generating sense and antisense single-stranded RNAs (ssRNAs). Equal amounts of sense and antisense ssRNAs were mixed and annealed according to our previous study [15]. The synthesized dsRNA was supplemented with 1 μL of RNase inhibitor, aliquoted into 10 μL volumes in 0.2 mL PCR tubes, and stored at −80 °C for later use. To eliminate non-specific effects of dsRNA, dsRNA targeting Enhanced Green Fluorescent Protein (*EGFP*; GenBank Accession Number: U55762.1) was employed as a negative control [16]. Primer sequences used for dsRNA synthesis are detailed in Table 1.

### 2.5. Functional Analysis of NinaB-like Gene Was Performed Using RNAi

In this study, embryos sourced from the same berried female were treated with *NinaB-like* dsRNA (treatment group, TG) or *EGFP* dsRNA (control group, CG). Each treatment contained at least 15 embryos. To facilitate the dsRNA into embryos, embryos were softened via exposure to 2% solution of HClO for 2 min. After being rinsed extensively (2–3 times, 5 min each) with sterile double-distilled water (ddH_2_O) to remove residual HClO, the embryos were then transferred to a new 24-well culture plates, with each well containing 1 mL of ddH_2_O. dsRNA was introduced to each well to achieve a final concentration of 5 μg/mL [17]. After a 2 h incubation period, the dsRNA-containing ultrapure water was removed, and embryos were washed twice with ddH_2_O. Embryos were then incubated for an additional 12 h in 1 mL of ddH_2_O. Embryonic morphological phenotypes were observed and documented using a Nikon SMZ25 stereomicroscope (Nikon Corporation, Tokyo, Japan).

The red pixel brightness ratio (RPB) of chromatophores was defined as the ratio of red pixel brightness to total pixel brightness. The RPB was quantified using Adobe Photoshop2020 software. The length and width of chromatophore clusters were measured using ImageJ (v1.54f) software. Pigment cell distribution state was quantified using the Pigment Distribution Scale (PDS) [18]. Statistical comparisons of phenotypic and gene expression levels between RNAi treatment and control groups were performed using *t*-tests [14]. Statistical significance was defined as *p* < 0.05. Pearson’s χ^2^-test was performed to investigate the relationships between SNP genotype and body color [19].

### 2.6. Candidate Gene Homology and SNP (Single-Nucleotide Polymorphism) Identification

For RNA sample preparation, the epidermis from 80 individual shrimps were pooled as one sample for total RNA extraction. The raw sequencing data have been deposited in the NCBI Sequence Read Archive (SRA) database under the integrated transcriptome accession number: SRX11349085 and SRX11349088. Raw sequencing reads were subjected to quality control using Trim Galore (v0.6.7) [20], which removed low-quality bases (consecutive bases with Phred quality score Q < 20), excluded reads with excessive ambiguous bases, and trimmed adapter sequences to generate clean reads. The QC-passed paired-end reads were merged via Pandaseq v2.11 [21] (minimum overlap length = 15 bp, sequence identity ≥ 90%) to yield consensus sequences in FASTA format.

The merged sequences were used for *de novo* transcriptome assembly via Trinity v2.15.1 [22] (k-mer = 25, minimum contig length ≥ 200 bp), generating a unigene set as the reference. Clean reads were aligned to this *de novo* transcriptome using Bowtie2 v2.4.6 [23], and uniquely mapped reads were filtered (MAPQ ≥ 20). Unmapping reads were re-assembled *de novo* (Trinity v2.15.1) to construct a supplementary transcriptome database; local alignment BLASTN v2.13.0 (E-value ≤ 1 × 10^−5^, alignment length ≥ 50 bp, identity ≥ 85%) was performed between validly mapped sequences and this supplementary database to clarify SNP locus homology [24].

SNP screening was conducted on the alignment results via SAMtools v1.17, and the identified SNPs were annotated using ANNOVAR (v20210601) [25] (associating SNPs with unigene structures: exonic/intronic regions; predicting effects: synonymous/nonsynonymous SNPs). The faSomeRecords tool (UCSC Genome Browser suite) [26] was used to extract SNP-flanking unigene sequences for context visualization. The mutant (minor) allele frequencies at each locus were defined as the ratio of mutant allele read counts to the total read counts.

The amino acid sequence of the candidate gene (Unigene0086585) obtained from cherry shrimp transcriptome assembly and PCR validation was used as the query sequence. Sequence similarity alignment was performed against the ninaB protein (a *BCO1* ortholog, GenBank accession no. NP_650307.2) from the model insect *Drosophila melanogaster* using the BLASTp tool (https://blast.ncbi.nlm.nih.gov/Blast.cgi, accessed on 14 May 2023) with default parameters. Meanwhile, the conservation of the core functional domain (RPE65 domain) was analyzed through multiple sequence alignment using ClustalX 2.1 software [27].

### 2.7. SNP Verification

In this method, the typing sites were captured in the first round of PCR, the barcode of the individual was added in the second round of PCR, the sequences of thousands of individuals at dozens of loci were mixed for second-generation high-throughput sequencing, and the genotype information of the individual loci was obtained via sequence comparison analysis.

#### 2.7.1. GT-Seq Primer Design

To prevent interference from introns in the genomic DNA (gDNA) template, genotyping primers must be designed to ensure that the amplified fragments are located within a single exon. To ensure compatibility with Illumina paired-end short-read Illumina sequencing technology and to guarantee sequencing quality and coverage, priority should be given to primer designs that position the SNP within the high-quality central sequencing region, specifically 50–100 bp from one of the primers. Additionally, the amplicon length should not exceed 300 bp. Target gene primer design software Primer 3 (https://sourceforge.net/projects/primer3/, accessed on 26 May 2023) was used to design primers. The specific steps for designing two rounds of primers are as follows:

In the first round of PCR, the primers were designed as target gene-specific sequences, with overhead adapter (OH1 for forward primer and OH2 for reverse primer) added to the 5′ end of each primer, respectively (Table 1). The first PCR was used to add OH1/OH2 adapters at the end of target gene fragments containing candidate SNP loci. The OH adapters served as binding sites for the second-round PCR primers. In the second PCR, indexed adapters (i5 and i7) were added to PCR products. The indexed adapter contains 6 bp barcodes for individual sample identification. A pair of indexed adapters marks one individual. A multiplexed approach was implemented using 8 ‘i5’ and 24 ‘i7’ tagging primers, generating 192 distinct barcode combinations (8 × 24) to uniquely identify each sample. The barcode sequences used in this study were obtained from the GT-seq protocol described previously [28].

#### 2.7.2. Genomic DNA Extraction

For SNP genotyping by Gt-seq (genotyping-in-thousands by sequencing), genomic DNA was isolated for 192 individual cherry shrimp from each strain via the Chelex 100. Briefly, 5.0 g of Chelex100 resin was accurately weighed into a 50 mL centrifuge tube, and 45 mL of sterile water was added to prepare a 10% Chelex100 solution. Subsequently, a total of 100 μL solution was pipetted into each well of a 96-well plate each containing 10–15 Chelex100 particles. After the cherry shrimp was anesthetized using ice according to Method 2.2, a single appendage was carefully dissected from each individual using sterile tweezers and transferred to the corresponding well of the 96-well plate. Once all samples were loaded, the 96-well plate was sealed with a silicone cap, verifying airtight closure of all wells. The plate was then centrifuged using a 96-well plate-specific centrifuge at 2800 rpm for 1 min to pellet the tissue at the bottom of the wells, facilitating complete tissue immersion in the Chelex solution. Subsequently, the plate was placed in a PCR thermocycler and subjected to 95 °C for 20 min. After the 96-well plate was removed from the PCR instrument, the plate was vortexed for 1 min. The supernatant contained genomic DNA of cherry shrimp, and 3 μL was taken as the first PCR template. If PCR amplification is not performed immediately, the 96-well plate can be stored at −20 °C with a maximum storage period of 1 month (PCR is preferably performed within 1 week), and a 1-min re-centrifugation is required prior to use.

#### 2.7.3. GT-Seq Library Construction

The library of GT-seq was constructed via two rounds of PCR [29]. In the first round of PCR amplification, the target sequence amplification primer with OH1/OH2 adapters was used, and the template was genomic DNA. The reaction profile for the first round of PCR is as follows: pre-denaturation at 95.0 °C for 5 min, followed by 35 cycles of denaturation at 95.0 °C for 30 s, annealing at 60.0 °C for 30 s, and extension at 72.0 °C for 20 s; this was then followed by a final extension at 72.0 °C for 10 min, and a hold at 4.0 °C for 5 min. Then, the amplification product was diluted 100 times and 3 μL was used as the template for the second amplification. The barcode with 6 unique bases was then used to further build the illumina sequencing library via the adapter above. The reaction profile for the second round of PCR was: pre-denaturation at 95.0 °C for 5 min, followed by 35 cycles of denaturation at 95.0 °C for 30 s, annealing at 70.0 °C for 30 s, and extension at 72.0 °C for 20 s; this was then followed by a final extension at 72.0 °C for 10 min, and a hold at 16.0 °C for 10 min. Finally, the second-round products of red strain and yellow strain were absorbed at 5 μL per hole for mixing sample delivery; blue strain and wild strain were also absorbed at 5 μL per hole for mixing sample delivery. The final library, containing 768 individuals, was sent to Novogene Co., Ltd. (Beijing, China), with sequencing using an Illumina NovaSeq 6000 instrument to perform pair-end sequencing (PE150, 2 × 150 bp) after sequencing library construction.

### 2.8. Data Analysis

For each target, sequences were merged into a single file. SNP loci were mapped based on the first-round primer binding sites, followed by base extraction via the mid function. Barcode loci were determined by locating second-round primer binding sites, followed by extraction of 5′ and 3′ barcodes (mid function) and concatenation into unique tag sequences. Based on the unique tag sequence of each individual, the number of occurrences of each SNP base was quantified. The allele frequency index (AFI) was defined as the ratio of the count of a specific allele to the total count of all alleles at the target SNP locus. Based on the AFI, individuals were classified as homozygous (AFI < 0.1 or AFI > 10), heterozygous (AFI < 0.2 or AFI > 5), or unclassifiable [13].

SNP locus correlations among the four strains were analyzed by Chi-square test using SPSS 22.0 [28]. RNA secondary structure alterations induced by SNPs were predicted using the mfold UNAFold online tool (http://www.unafold.org/mfold/applications/rna-folding-form-v2.php, accessed on 23 March 2024).

## 3. Results

### 3.1. NinaB-like Expression Profiles

**Figure 1:**
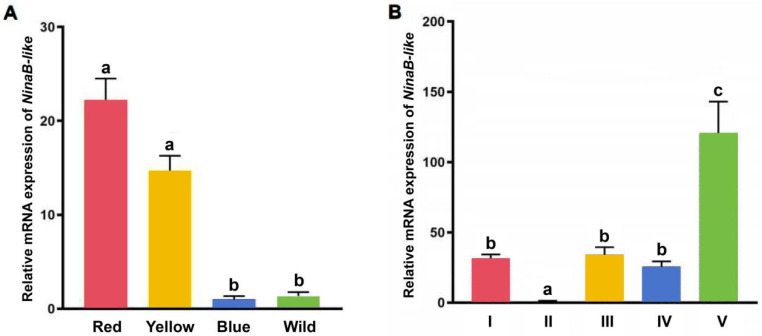
Relative expression of *NinaB-like* in red cherry shrimp. (**A**) Strain-specific expression profiles of *NinaB-like*; (**B**) Developmental stage expression patterns of *NinaB-like* in the red strain. I: pre-nauplius stage; II: metanauplius stage; III: pre-zoea stage; IV: membrane-zoea stage; V: post-larva stage. Different lowercase letters above the bars indicate significant differences among groups (*p* < 0.05). Error bars represent the mean ± standard error (SE). All experiments were performed with seven technical replicates.

Quantitative analysis revealed significantly elevated *NinaB-like* expression in red and yellow strains compared to blue and wild-type strains (*p* < 0.05), while no statistically significant differential expression was observed between red and yellow strains (*p* > 0.05) (Figure 1A).

Developmental stage profiling demonstrated that post-larva *NinaB-like* expression level significantly exceeded that of all other stages (*p* < 0.05). Conversely, the metanauplius stage exhibited markedly reduced expression levels relative to other developmental stages (*p* < 0.05) (Figure 1B).

### 3.2. Functional Analyses of NinaB-like Gene via RNA Interference (RNAi)

**Figure 2:**
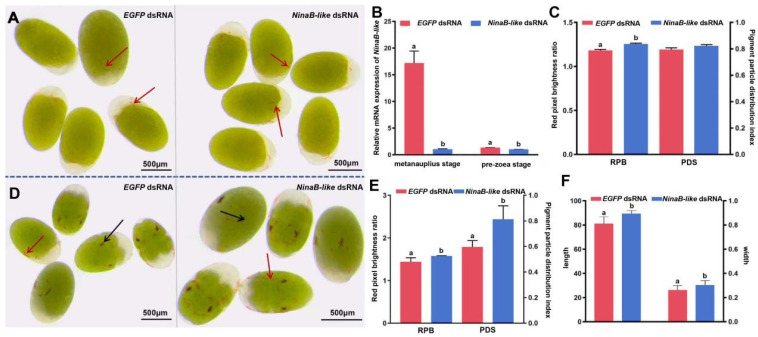
Effects of *NinaB-like* interference on embryonic morphology and related indicators in cherry shrimp. (**A**) Phenotypic changes after *NinaB-like* dsRNA treated larvae at metanauplius stage; (**B**) The relative expression of *NinaB-like* after interference in the red strain; (**C**) The RPB and the PDS of metanauplius. The group of bars on the left represents RPB, the group of bars on the right represents PDS; (**D**) Phenotypic changes after *NinaB-like* dsRNA treated larvae at pre-zoea stage; Chromatophore clusters within the developing compound eyes are indicated by black arrows, while erythrophores are denoted by red arrows. (**E**) The RPB and the PDS of pre-zoea. The group of bars on the left represents RPB, the group of bars on the right represents PDS; (**F**) The length and width of chromatophore cluster in compound eyes. Embryos were derived from the same female. Statistically significant differences (*p* < 0.05) are represented by different lowercase letters. Error bars represent the mean ± SE.

Phenotypic analysis demonstrated that *NinaB-like* dsRNA-mediated knockdown at the metanauplius stage resulted in significantly enhanced pigmentation intensity within chromatophores relative to the control group (Figure 2A). After RNAi, qPCR analysis revealed significant downregulation of target gene transcripts in the treatment group (TG) compared to the control group (CG) (*p* < 0.05) (Figure 2B), and the RPB of pigment cells was significantly higher than the *EGFP* dsRNA of the control group (*p* < 0.05) (Figure 2C). The morphology of pigment cells in the treatment group was similar to that in the CG, and there was no significant difference in pigment particle distribution index (*p* > 0.05) (Figure 2C).

The condition of the pre-zoea stage after RNAi was similar to the metanauplius stage. The color of pigment cells in the TG was significantly deepened (Figure 2D), and the RPB and PDS of pigment cells was significantly higher than the CG (*p* < 0.05) (Figure 2E). The length and width of compound eyes pigment cells in TG were significantly higher than in CG (*p* < 0.05) (Figure 2F).

### 3.3. NinaB-like Homology Identification, Genotyping and Correlation Analyses

**Figure 3:**
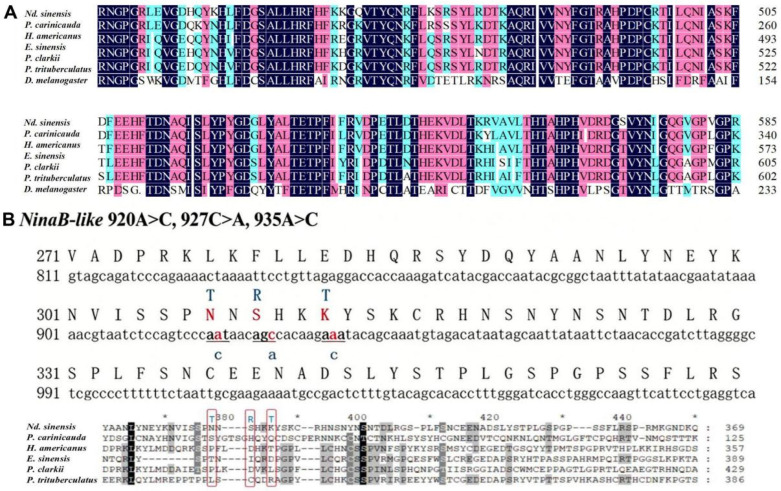
Partial sequence variations of *NinaB-like*. (**A**) Partial conserved functional domain sequence of RPE65. The background color intensity indicates the degree of residue conservation: darker colors denote positions that are highly conserved across all aligned species; lighter colors denote positions conserved in only a subset of species. (**B**) The sequence near the *NinaB-like* mutation site. Capital letters represent amino acids, lowercase letters represent nucleotides, red letters represent reference amino acids or nucleotides, blue letters represent alternative amino acids or nucleotides, and triplet codons are underlined. The short black line represents a single amino acid encoded by three consecutive codons (corresponding to the underlined triplet codon in the upper panel). The annotated region (red box) displays the polymorphic amino acid positions resulting from SNPs in cherry shrimp and other species. The background color intensity indicates the degree of amino acid conservation, with darker shades representing higher conservation levels. The * is used to evenly separate the amino acid sequence.

Sequence similarity analysis demonstrated that the *NinaB-like* gene identified in this study exhibits 41.99% amino acid sequence identity to the ninaB protein (GenBank accession number NP_650307.2) from the model insect *Drosophila melanogaster*. The high conservation of the core RPE65 functional domain further confirms that this gene encodes a BCO1 family homolog in cherry shrimp (see supplementary annotation in Figure 3A).

The identification, quality control screening of SNPs and calculation of *F_st_* values were performed as described in our previous publication [30]. There were three candidate SNPs in *NinaB-like*, named C.920A > C, C.927C > A, C.935A > C. 920A > C, which had no polymorphisms among different strains of cherry shrimp, so they are not shown here.

C.927C > A (*NinaB-like*) and C.935A > C (*NinaB-like*) were the third and second codons, respectively, resulting in 309S > R and 312K > T (Figure 3B). Amino acids at positions 309 and 312 were also less conservative, with H, D, I, and C residues appearing in the former and Q, T, L, and R residues appearing in the latter (Figure 3B). Other species do not show the mutant amino acid R in cherry shrimp at position 309, and *Homarus americanus* had the same amino acid residue T at position 312 as cherry shrimp.

**Figure 4:**
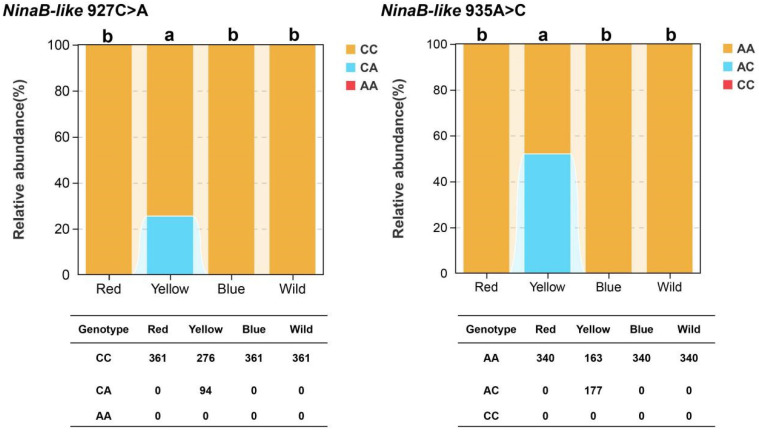
Genotype frequencies of *NinaB-like* SNPs polymorphisms in different color strains. Bar plots represent the relative abundance of each genotype. The table below each plot shows the genotype counts of each strain. Different letters above the bars indicate statistically significant differences in genotype frequencies among strains (*p* < 0.05).

Only the yellow strain showed heterozygosity in these two SNP genotypes (CA and AC), and the other three strains showed no polymorphisms (Figure 4). There was a significant genotype frequency between the yellow strain and the other strains (*p* < 0.05).

**Figure 5:**
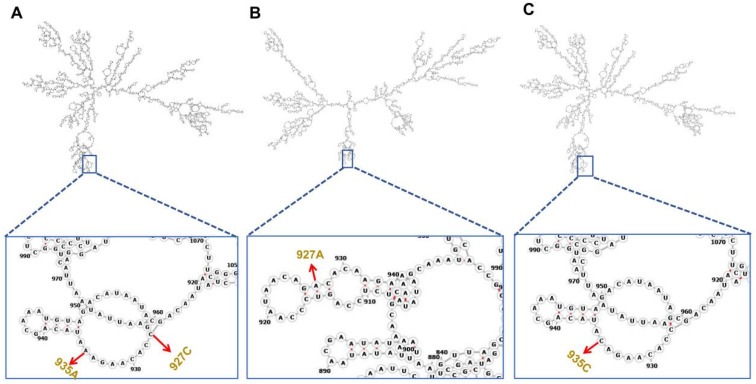
Effect of SNPs on RNA secondary structure. (**A**) Primitive RNA secondary structure of *NinaB-like*; (**B**) 927C > A RNA secondary structure of *NinaB-like*; (**C**) 935A > C RNA secondary structure of *NinaB-like.* The black line represents the RNA secondary structure, with colored boxes indicating magnified views of the corresponding boxed regions. The black capital letter represents an amino acid. Red arrows indicate the precise location of the SNP.

The secondary structure of mRNA before mutation was characterized by a compact hairpin loop near the 927/935 region (Figure 5A). When the 927C > A mutation occurred (Figure 5B), the local secondary structure around this site was significantly altered: the original hairpin loop was disrupted, and a new single-stranded region formed at the 927A locus. Similarly, the 935A > C mutation (Figure 5C) induced a conformational change in the adjacent loop structure, with the 935C allele leading to a more relaxed RNA folding pattern in this region.

## 4. Discussion

Reducing carotenoid oxygenase activity is a well-established strategy to enhance carotenoid accumulation in aquatic animals [31,32]. In crustaceans, diminished expression or genetic ablation of the BCO1 family in *Eriocheir sinensis* [31] and *Exopalaemon carinicauda* [33,34] resulted in a significant increase in both the redness value and β-carotene content within the hepatopancreas. These studies consistently indicate that BCO1 family genes act as negative regulators of carotenoid deposition, and their functions are highly conserved among vertebrates and invertebrates. Our study confirmed that *NinaB-like*, a *BCO1* homolog, functions as a negative regulator of carotenoid deposition in cherry shrimp. Specifically, *NinaB-like* was highly expressed in the red strain and post-larval stage, where chromatophores are abundant. This suggests that *NinaB-like* mediates carotenoid homeostasis to adapt to high pigment accumulation in red shrimp, as supported by our unpublished LC-MS data showing peak carotenoid levels in the red strain [8].

More direct evidence comes from RNAi experiments: significantly deeper RPB and PDS values in the experimental groups of larvae. This phenotype is completely consistent with the enhanced pigment accumulation phenotype observed after knockout of the *BCO1* family genes in crustaceans [31,33,34], clearly confirming that the *NinaB-like* gene in cherry shrimp also has the negative regulatory function of promoting carotenoid degradation. Notably, there are subtle differences in the regulatory mechanisms of *BCO1* family genes among both vertebrates and invertebrates [35,36,37]. These differences may be the result of long-term adaptation of species to different niches, but the core negative regulatory function is highly conserved. Combined with the cross-species conserved functional background of *BCO1* family genes and the species-specificity of regulatory mechanisms, the expression characteristics and interference phenotypes of the *NinaB-like* gene in cherry shrimp in this study further enrich our understanding of the conserved mechanism of carotenoid metabolism regulation in crustaceans.

Two non-synonymous SNPs (927C > A and 935A > C) in *NinaB-like* were strongly associated with body color, with heterozygotes exclusively detected in the yellow strain. SNPs in *BCO1* orthologs are known to alter carotenoid metabolism in various animals [38,39,40,41]. In cherry shrimp, the 927C > A (S309R) substitution introduces a charge change (serine→arginine) at a poorly conserved site. Based on the conserved functional characteristics of *BCO1* family genes and the above-mentioned mutation effects, we hypothesize that these two SNPs in the yellow strain jointly reduce the carotenoid cleavage activity of the NinaB-like enzyme, leading to β-carotene accumulation. This accumulated β-carotene further imparted the yellow body color phenotype to the cherry shrimp, which is consistent with our previous findings that the yellow strain has significantly higher β-carotene content compared to the blue and wild strains [8].

## 5. Conclusions

Our findings fill a critical gap in understanding crustacean color regulation by providing the first functional evidence for *NinaB-like* in cherry shrimp. Unlike mammalian and insect *BCO1* homologs [42,43], *NinaB-like* in cherry shrimp directly links carotenoid degradation to body color polymorphism, offering a molecular target for ornamental shrimp breeding. Future research could use CRISPR-Cas9 to validate SNP functions [34] and explore interactions between *NinaB-like* and other carotenoid metabolic genes (e.g., *BCO*, *NinaB*) [31], as recently reported in aquatic invertebrates.

## Figures and Tables

**Table 1:** The PCR primers used in this study.

Primer Name	Primer Sequences (5′-3′)	Application
NinaB-like Fq	AACGAATATAAAAACGTAATCTCCAG	qPCR
NinaB-like Rq	GAGCCCCTAAGATCGGTGT	qPCR
GADPH Fq	CGGTGCTGCTCAGAATATCA	qPCR
GADPH Rq	TTACCAAGGCGAACGGTAAG	qPCR
NinaB-like Fd	CATCCTGAAGCACAGAACGA	dsRNA generation
NinaB-like Rd	CGTATCTCCCTCCTTCCCTC	dsRNA generation
dsEGFP F	GGTGAACTTCAAGATCCGCC	dsRNA generation
dsEGFP R	CTTGTACAGCTCGTCCATGC	dsRNA generation
T7F	TAATACGACTCACTATAGGG	Adapter
T7R	CCCTATAGTGAGTCGTATTA	Adapter
NinaB-like F1	AACGAATATAAAAACGTAATCTCCAG	Target sequence amplification
NinaB-like R1	AGCCCCTAAGATCGGTGT	Target sequence amplification
OH1	CGACAGGTTCAGAGTTCTACAGTCCGACGATC	Add adapter
OH2	GCTCGTCGTGACGCCATGACGG	Add adapter

Note: All primers were designed based on the following gene sequences with GenBank accession numbers: *NinaB-like* (PRJNA1209659), *GAPDH* (MZ734609), *EGFP* (U55762.1). T7 promoter and OH1/OH2 adapters are universal sequences without specific GenBank accession numbers.

## Data Availability

The original contributions presented in this study are included in the article. Further inquiries can be directed to the corresponding author.
